# Causes of Discoloration in Gold Fillings in Teeth

**Published:** 1884-12

**Authors:** William Herbert Rollins


ARTICLE IV.

### CAUSES OF DISCOLORATION IN GOLD FILLINGS IN THE TEETH.

BY WILLIAM HERBERT ROLLINS, D. D. S.

As pure gold is not supposed to discolor, the causes must be other substances which may be introduced in one or more of several ways: they may be present in or on the gold foil when received from the makers; they may be present in the mouth itself.

The best way to begin the investigation was evidently to collect the discolored portions of fillings and analyze them. As yet no satisfactory result has been reached in this way, because the quantities were too minute.

The fillings themselves were next examined. Iron, silver, tin, and mercury were found. In which of the ways mentioned did these metals get into the fillings? The first attempts made to answer this question were to get samples of gold foil from the prominent makers, number them, and send them to Dr. Hills, of the Harvard Medical School, to be analyzed. The result of these analyses showed that none of the samples were pure. This was unexpected, as the makers guarantee their foils to be one thousand fine, or absolutely pure. Silver was found in every sample and iron in some. Extensive analyses made by the same chemist a year later showed the same results. The gold prepared by R. S. Williams always contained the least impurities. As the gold when received from the makers was always found impure, a short description of the manufacture may be of value as showing how impurities may be introduced.

The gold out of which foil is made is the mint gold, containing about two parts in a thousand of impurities. The makers dissolve this in aqua regia, filter, precipitate with ferrous sulphate, or more rarely with oxalic acid, wash the precipitate, melt it, and after casting, roll to ribbons between steel cylinders. It is then beaten to the required thickness between pieces of glued skin, which are never clean.

Gold in this form I shall call plain foil, to distinguish it from the forms in use. Plain foil is not in the market. It must be obtained by special order. To get this plain foil into the numerous varieties required by those who fill teeth. it passes through several processes and then becomes soft gold, cohesive gold, gold cylinders, gold blocks, gold pellets, etc., etc.; indeed one maker advertises over fifty varieties.

Cohesive Gold. Plain foil is heated (usually on an iron plate.)

Soft Gold. I am not sure how this is made, as makers of foil have their secrets, but as surface impurities have been found on this kind of toil it is probable that, in some cases, the softness is due to those impurities which prevent two surfaces of this gold from uniting, and thus produce the so-called softness.

Corrugated Foil. Alternate sheets of gold and thin paper are made into a pile, placed between two pieces of iron and heated in a furnace till the paper is consumed. The sheets of gold are then separated, the ash of the paper removed as much as possible, after which the leaves are placed in books, or used in the manufacture of gold blocks, gold cylinders, gold ribbons, etc., etc.,

Gold Blocks, Gold Cylinder, etc. These are the forms of gold in most general use. To make them the foil (usually the corrugated kind) is subjected to various rolling, pressing, and cutting processes by iron machinery.

This brief description of the manner of making gold for dental use enables us to see how easily impurities may be present in the finished product.

Silver, which is always present in the gold before refining, is almost sure to be present after the process, through careless manipulation and poor acids. Iron is likely to be introduced from the ferrous sulphate, from the moulds, from the steel rollers, from the plates on which the gold is heated, from the hands, from the machinery used to form it into pellets, cylinders, etc. Other impurities are almost certain to come from the skins used in beating, from the paper used in corrugating, and from the dust of the shop.

To test whether impurities could be introduced by a careful person in making fillings, the following experiments were tried. The foil used was plain foil, and contained no impurities except a small amount of silver, as shown by a very careful analysis by Dr. Hills :—

First experiment. The foil was prepared in the ordinary way, as follows : It was cut with steel scissors, heated on iron, handled with steel tweezers, condensed with serrated steel pluggers, burnished with a steel burnisher. Dr. Hill's analysis showed that iron was present in these fillings.

Second experiment. The same foil was treated as before, only iridium instruments, made by the American Iridium Company of Cincinnatti, were used instead of steel. Iron was found in these fillings.

Third experiment. The same foil was cut with bamboo handled with gold tweezers, heated on platinum and condensed with iridium points, and burnished with an agate burnisher. No iron was found in these fillings.

This brief description can give no idea of the amount of care and time which has been spent on these investigations. I give a short abstract from one of Hill's reports, but even this conveys no adequate idea of the amount of delicate work in the analyses:—

"It is proper that I should state that great pains have been taken in the analyses to arrive at correct results. In the beginning great difficulty was experienced in obtaining chemicals of the requisite purity, and it was only after considerable time and trouble that acids absolutely pure were secured. In order to make certain that, if any of the metals tested for were found, they were contained in the gold itself, bland analyses were made before each analysis of the gold, using the same apparatus and chemicals."

Thus far the only metals whose presence we have accounted for are iron and silver. The next question which arises is, How came the tin and mercury in the fillings in which they were found?

Almost every dentist now uses amalgam in greater or less quantity. This remains on the instruments in minute amounts, often invisible; and when, by accident or otherwise, the same burnishers perhaps are used in making a gold filling, a little of the amalgam gets upon the surface, showing itself after a time as a discolored filling. That this is a frequent cause of discoloration I have no doubt, and only by performing all amalgam operations in another room with an entirely separate set of instruments can we hope to get rid of the trouble. As an illustration, I should like to mention the following case: An amalgam filling had been made in the morning, the hands carefully washed, and other fillings of gold made before lunch. In the afternoon a large gold filling had been almost completed, but needed a little burnishing. The gold burnisher was moistened with soap and applied to the filling, which directly changed color. An examination of the burnisher showed the same trouble. For a long time I was unable to account for the mischief. At last it was traced to the soap, to which a little of the amalgam must have clung when the hands were washed in the morning after using the amalgam.

In the beginning of the paper three ways were suggested in which impurities might get into gold fillings. I have shown that in the first and second of these ways impurities are introduced, and with regard to the third way the following may be said: Certain medicines, as iron, when allowed to remain in contact with the teeth, cause a discoloration of the debris which collects about the fillings and on their surfaces. In the mouth of those who smoke, the deposits of epithelial cells mixed with lime and germs which collect about imperfectly cleaned fillings become darkened. This discoloration makes fillings in some cases almost black. Discolored fillings are due to many different causes, as I have shown. In conclusion, the following suggestions, if faithfully carried out, will do away with some of these causes:—

Demand purer gold from the makers. Use this in the form of plain foil, because this is least liable to contamination.

Never touch gold foil with steel instruments of any kind. If it is necessary to heat the gold, do this on platinum.

Keep a separate set of instruments for amalgam. Insist upon a thorough cleaning of the mouth every time food or medicines are taken.—*Boston Med. and Surg. Journal.*

